# Supplementary material for: Ingredients for microbial life preserved in 3.5 billion-year-old fluid inclusions
Source: Nat Commun. 2021 Feb 17;12:1101. doi: 10.1038/s41467-021-21323-z (PMC7889642; doi:10.1038/s41467-021-21323-z)
Supplement: Supplementary file 1 — Supplementary Information [file 41467_2021_21323_MOESM1_ESM.pdf]

Supplementary information

Ingredients for microbial life preserved in 3.5 billion-year-old fluid inclusions

Helge Mißbach et al.

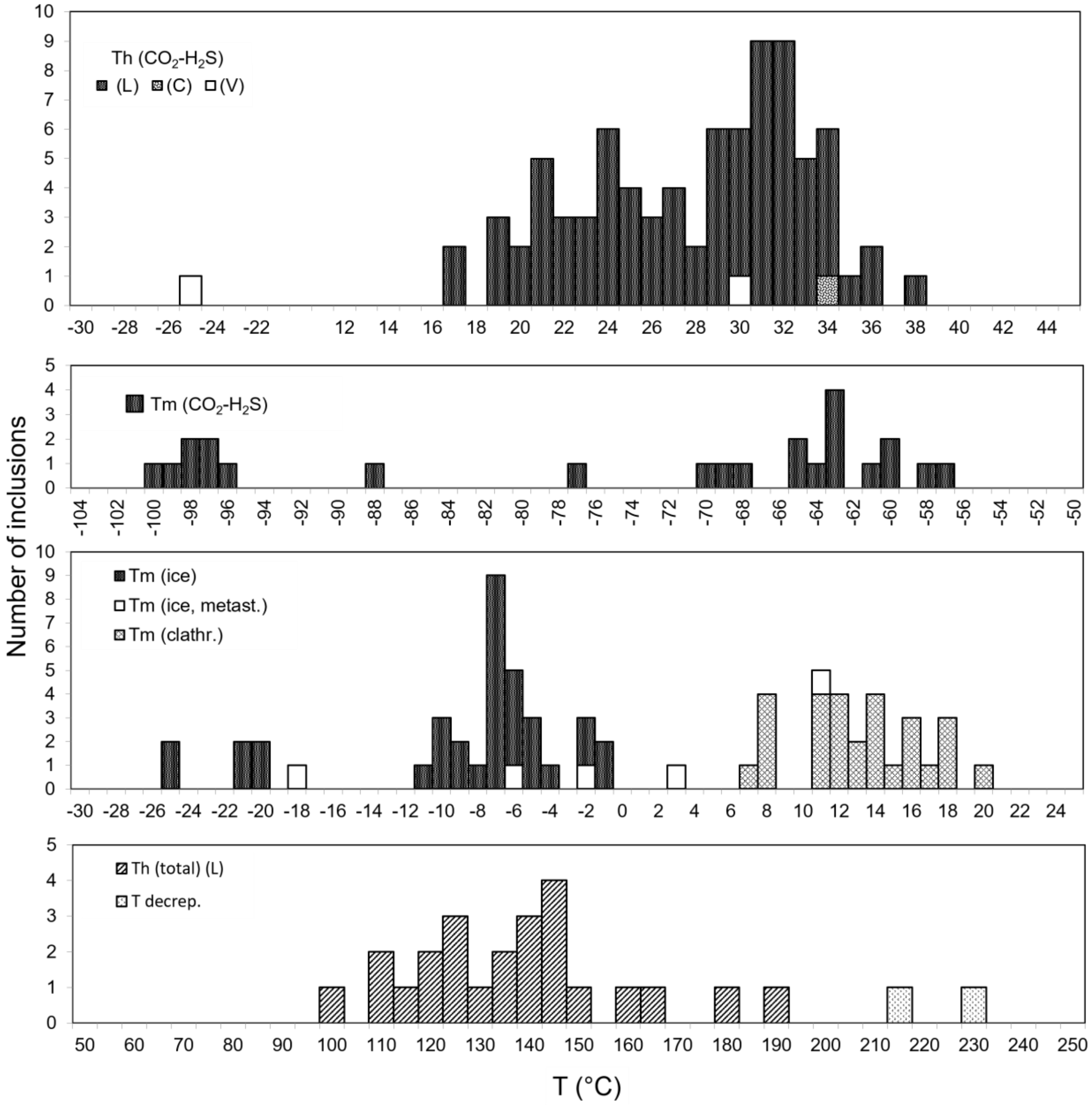

Supplementary Figure 1: Bar plots showing phase transition temperatures. From top to bottom: (i) homogenisation of the non-aqueous phase [Th (CO_2-H_2S)], (ii) melting of CO_2 and H_2S [Tm (CO_2-H_2S)], (iii) ice and clathrate melting [Tm (ice)/Tm (ice, metast.) and Tm (clathr.), respectively], (iv) total homogenization and decrepitation [Th (total) and T decrep., respectively] temperatures. C, critical; V, vapour, L, liquid.

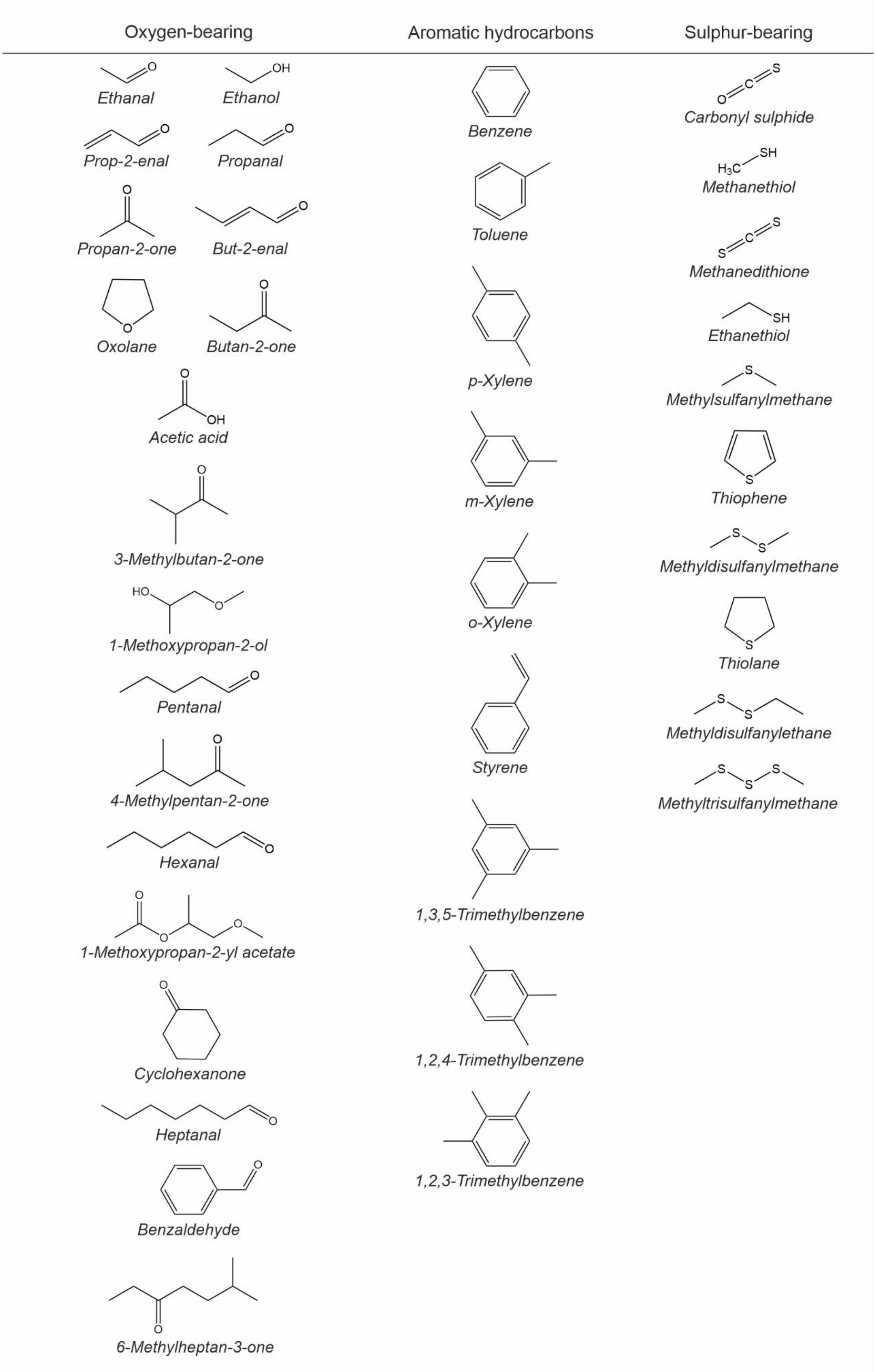

Supplementary Figure 2: Molecular structures of oxygen-bearing compounds, aromatic hydrocarbons, and sulphur-bearing compounds found in black barite fluid inclusions.

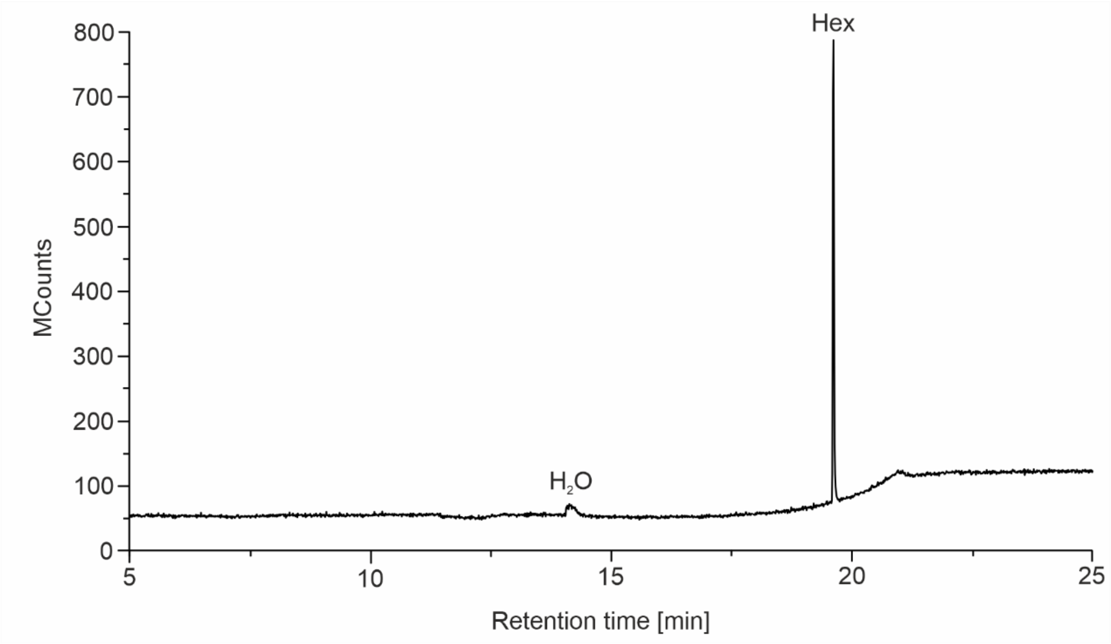

Supplementary Figure 3: Total ion current (TIC) chromatogram of thermal decrepitation/desorption GC–MS (TD-GC–MS) pre-analysis blank obtained at 250°C. Hex = *n*-hexane (retention time standard).

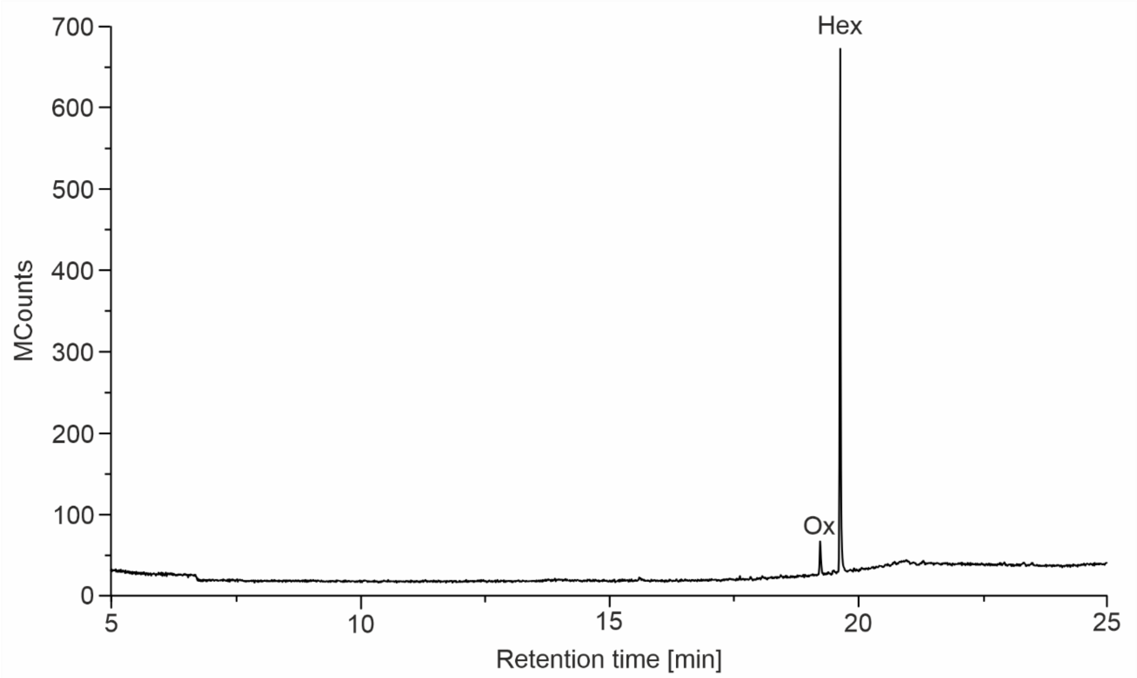

Supplementary Figure 4: Total ion current (TIC) chromatogram of solid phase micro extraction GC-MS (SPME-GC–MS) pre-analysis blank (uncrushed sample in grinding jar). Ox = oxolane; Hex = *n*-hexane (retention time standard).

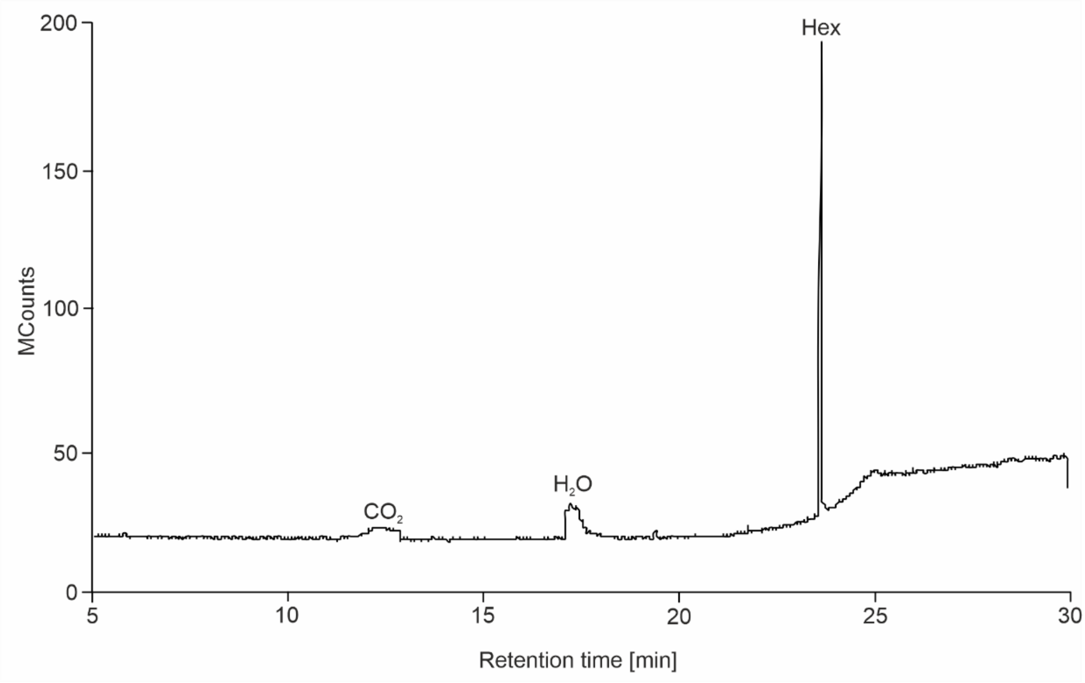

Supplementary Figure 5: Total ion current (TIC) chromatogram of volatile compounds released from white barite (very lean in fluid inclusions) by thermal decrepitation/desorption GC–MS (TD-GC–MS) obtained at 250°C. Note that only CO_2 and H_2O were observed in minor abundances. This analysis serves as negative control. Hex = *n*-hexane (retention time standard).

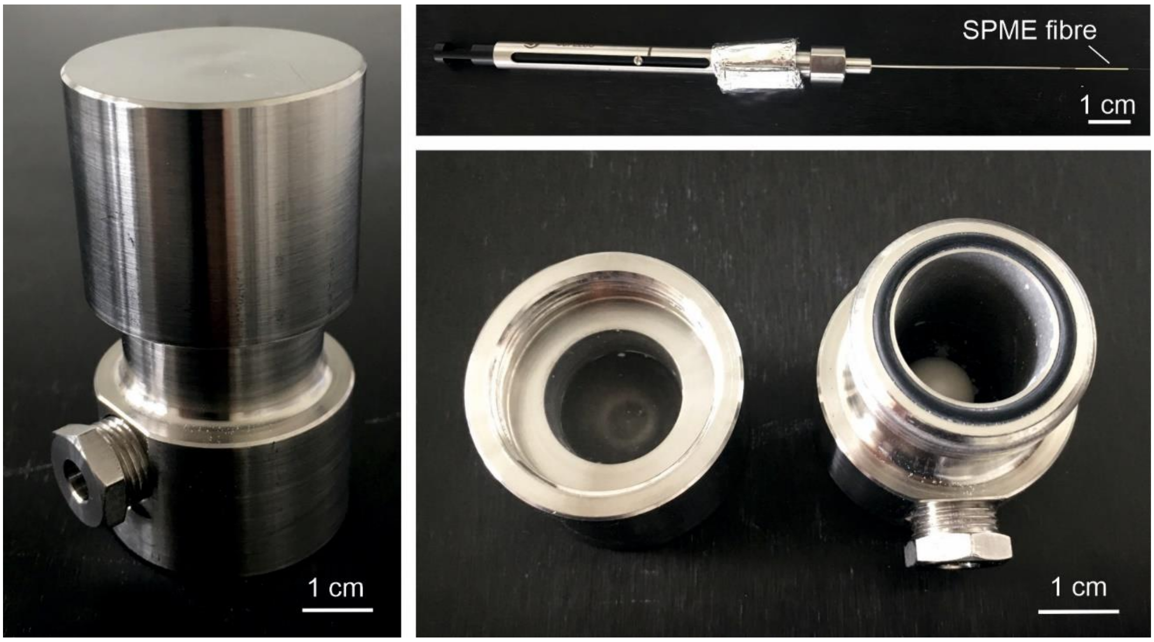

Supplementary Figure 6: Grinding jar equipped with a standard septum port and solid phase micro extraction (SPME) syringe with fibre exposed (upper right).
