# Supplementary material for: Ingredients for microbial life preserved in 3.5 billion-year-old fluid inclusions
Source: Nat Commun. 2021 Feb 17;12:1101. doi: 10.1038/s41467-021-21323-z (PMC7889642; doi:10.1038/s41467-021-21323-z)
Supplement: Supplementary file 3 — Reporting Summary [file 41467_2021_21323_MOESM3_ESM.pdf]

nature research

Corresponding author(s): Helge Mißbach
University of Cologne

Last updated by author(s): Nov 17, 2020

Reporting Summary

Nature Research wishes to improve the reproducibility of the work that we publish. This form provides structure for consistency and transparency in reporting. For further information on Nature Research policies, see our Editorial Policies and the Editorial Policy Checklist.

Statistics

For all statistical analyses, confirm that the following items are present in the figure legend, table legend, main text, or Methods section.

n/a	Confirmed	
☒	☐	The exact sample size (*n*) for each experimental group/condition, given as a discrete number and unit of measurement
☒	☐	A statement on whether measurements were taken from distinct samples or whether the same sample was measured repeatedly
☒	☐	The statistical test(s) used AND whether they are one- or two-sided<br>*Only common tests should be described solely by name; describe more complex techniques in the Methods section.*
☒	☐	A description of all covariates tested
☒	☐	A description of any assumptions or corrections, such as tests of normality and adjustment for multiple comparisons
☒	☐	A full description of the statistical parameters including central tendency (e.g. means) or other basic estimates (e.g. regression coefficient) AND variation (e.g. standard deviation) or associated estimates of uncertainty (e.g. confidence intervals)
☒	☐	For null hypothesis testing, the test statistic (e.g. *F*, *t*, *r*) with confidence intervals, effect sizes, degrees of freedom and *P* value noted<br>*Give P values as exact values whenever suitable.*
☒	☐	For Bayesian analysis, information on the choice of priors and Markov chain Monte Carlo settings
☒	☐	For hierarchical and complex designs, identification of the appropriate level for tests and full reporting of outcomes
☒	☐	Estimates of effect sizes (e.g. Cohen's *d*, Pearson's *r*), indicating how they were calculated

Our web collection on statistics for biologists contains articles on many of the points above.

Software and code

Policy information about availability of computer code

Data collection	No code was used to collect data in this study.
Data analysis	No code was used to analyze the data in this study.

For manuscripts utilizing custom algorithms or software that are central to the research but not yet described in published literature, software must be made available to editors and reviewers. We strongly encourage code deposition in a community repository (e.g. GitHub). See the Nature Research guidelines for submitting code & software for further information.

Data

Policy information about availability of data

All manuscripts must include a data availability statement. This statement should provide the following information, where applicable:
- Accession codes, unique identifiers, or web links for publicly available datasets
- A list of figures that have associated raw data
- A description of any restrictions on data availability

All relevant data to support the findings are given in the paper (results section & Figs. 1 - 6) and the supplementary file. GC-MS raw data are available from the corresponding author upon request.

Field-specific reporting

Please select the one below that is the best fit for your research. If you are not sure, read the appropriate sections before making your selection.

☐ Life sciences ☐ Behavioural & social sciences ☒ Ecological, evolutionary & environmental sciences

For a reference copy of the document with all sections, see nature.com/documents/nr-reporting-summary-flat.pdf

Ecological, evolutionary & environmental sciences study design

All studies must disclose on these points even when the disclosure is negative.

Study description	Fluid inclusions in black barites were investigated for their volatile organic content.
Research sample	Black barites were used for (organic) geochemical analyses as they contained fluid inclusions with organic matter visible by petrographic analyses. All relevant information is given in the manuscript.
Sampling strategy	Different kinds of barites containing different amounts of fluid inclusions were collected (white, grey, black). No statistical methods were used for sampling in this geological study.
Data collection	Data was collected by means of Raman spectroscopy, fluid inclusion microanalysis, gas chromatography-mass spectrometry and stable isotope mass spectrometry by the corresponding author and co-authors (see author contributions section in the manuscript). No statistical data collection was performed in this geological study.
Timing and spatial scale	Not relevant for this geological study.
Data exclusions	No data were excluded.
Reproducibility	Different geochemical analyses were carried out at least twice. Results matched in every attempt.
Randomization	Not relevant for this geological study.
Blinding	Not relevant for this geological study.

Did the study involve field work? ☒ Yes ☐ No

Field work, collection and transport

Field conditions	Sunny, >30°C
Location	Abandoned barite Mine, Pilbara, Western Australia (21° 09' 05.2'' S, 119° 26' 15.3'' E)
Access & import/export	The abandoned part of the barite mine was accessed by car on gravel roads. Samples were taken from a recent cut wall and transported to the car on foot.
Disturbance	No disturbance was caused by this study.

Reporting for specific materials, systems and methods

We require information from authors about some types of materials, experimental systems and methods used in many studies. Here, indicate whether each material, system or method listed is relevant to your study. If you are not sure if a list item applies to your research, read the appropriate section before selecting a response.

Materials & experimental systems

n/a	Involved in the study	
☒	☐	Antibodies
☒	☐	Eukaryotic cell lines
☐	☒	Palaeontology and archaeology
☒	☐	Animals and other organisms
☒	☐	Human research participants
☒	☐	Clinical data
☒	☐	Dual use research of concern

Methods

n/a	Involved in the study	
☒	☐	ChIP-seq
☒	☐	Flow cytometry
☒	☐	MRI-based neuroimaging

Palaeontology and Archaeology

Specimen provenance	The barite samples containing small stromatolite layers were collected 2017 in an abandoned barite Mine (Pilbara, Western Australia, 21° 09' 05.2'' S, 119° 26' 15.3'' E) which is not a geoheritage site. Therefore, no permits were required.
Specimen deposition	University of Göttingen, Geoscience Centre, Geobiology division
Dating methods	No new dates are provided in this study.

☐ Tick this box to confirm that the raw and calibrated dates are available in the paper or in Supplementary Information.

Ethics oversight	No ethical approval or guidance was required as a) the stromatolite layers which are associated with the sampled barites are not a protected specimen and b) the sampling site is not a geoheritage site.

Note that full information on the approval of the study protocol must also be provided in the manuscript.
